# Corneal injury is associated with stromal and vascular alterations within cranial dura mater

**DOI:** 10.1371/journal.pone.0284082

**Published:** 2023-04-20

**Authors:** Olga V. Glinskii, Vladislav V. Glinsky, Leike Xie, Filiz Bunyak, Vladimir V. Glinskii, Sunilima Sinha, Suneel Gupta, Renato V. Iozzo, Rajiv R. Mohan

**Affiliations:** 1 Department of Medical Pharmacology and Physiology, University of Missouri, Columbia, MO, United States of America; 2 Dalton Cardiovascular Research Center, University of Missouri, Columbia, MO, United States of America; 3 Harry S. Truman Memorial Veterans’ Hospital, Columbia, MO, United States of America; 4 Department of Pathology and Anatomical Sciences, University of Missouri, Columbia, MO, United States of America; 5 Department of Electrical Engineering and Computer Science, University of Missouri, Columbia, MO, United States of America; 6 Department of Pulmonary, Allergy, and Critical Care Medicine, Stanford University Medical Center, Stanford, CA, United States of America; 7 Departments of Veterinary Medicine & Surgery and Biomedical Sciences, College of Veterinary Medicine, University of Missouri, Columbia, MO, United States of America; 8 Department of Pathology, Anatomy, and Cell Biology, The Translational Cellular Oncology Program, Sidney Kimmel Medical College at Thomas Jefferson University, Philadelphia, Pennsylvania, United States of America; 9 Mason Eye Institute, School of Medicine, University of Missouri, Columbia, MO, United States of America; Eötvös Loránd Research Network Biological Research Centre, HUNGARY

## Abstract

The cornea and cranial dura mater share sensory innervation. This link raises the possibility that pathological impulses mediated by corneal injury may be transmitted to the cranial dura, trigger dural perivascular/connective tissue nociceptor responses, and induce vascular and stromal alterations affecting dura mater blood and lymphatic vessel functionality. In this study, using a mouse model, we demonstrate for the first time that two weeks after the initial insult, alkaline injury to the cornea leads to remote pathological changes within the coronal suture area of the dura mater. Specifically, we detected significant pro-fibrotic changes in the dural stroma, as well as vascular remodeling characterized by alterations in vascular smooth muscle cell (VSMC) morphology, reduced blood vessel VSMC coverage, endothelial cell expression of the fibroblast specific protein 1, and significant increase in the number of podoplanin-positive lymphatic sprouts. Intriguingly, the deficiency of a major extracellular matrix component, small leucine-rich proteoglycan decorin, modifies both the direction and the extent of these changes. As the dura mater is the most important route for the brain metabolic clearance, these results are of clinical relevance and provide a much-needed link explaining the association between ophthalmic conditions and the development of neurodegenerative diseases.

## Introduction

Cranial dura mater (DM), a highly innervated and vascularized outermost meningeal layer, represents the most important site for the brain venous blood and cerebrospinal fluid (CSF) outflow. Compromised dura vascular integrity within both blood and lymphatic vessels conceivably contributes to the development of neurological disorders through the impairment of the brain metabolic waste clearance and alteration of homeostatic functions [1–3]. The involvement of dura mater lymphatics in neurodegeneration has already been demonstrated [4]. It has been shown that dysregulated meningeal lymphatic drainage results in decreased clearance of toxic proteins and accumulation of β-amyloid [5] and α-synuclein [6] in the brain leading to the development of impaired brain functionality.

Anatomical studies have shown that dura mater vasculature receives sensory innervation from the trigeminal nerve originating from the ophthalmic part of the trigeminal ganglion (TG) [7], which also innervates the cornea, the most sensitive part of the eye. Sensory innervation of the cornea and DM from the same TG ophthalmic division may provide a potential link between TG activation mediated by corneal injury and DM nociceptor sensitization, which in turn triggers vascular and stromal alterations within the cranial dura.

According to several studies, a link between ophthalmic conditions (including cataract and diabetes-related eye disease) and cognitive impairment have been suggested [7]. Shang X. et al. [8] showed that these ophthalmic conditions were independently associated with an increased risk of dementia from any cause compared to individuals without these conditions. Neuroimaging investigations, using functional magnetic resonance imaging, demonstrated that noxious stimulation of the cornea can produce somatotopic activation in the primary somatosensory cortex [9]. It has been suggested that any injury activating corneal nociceptors may trigger cellular, molecular, and functional changes within trigeminal ganglion and the central nervous system [10]. Particularly, alkali injury could cause corneal pain activating the neuropathic pain matrix in the CNS [11]. However, it is unclear whether ophthalmic conditions, including corneal injury, can induce vascular intracranial changes, particularly within cranial dura mater.

The underlying hypothesis for the present study is that pathological events occurring in the cornea can impact distant vascular cells and the extracellular matrix (ECM) within dura mater blood and lymphatic system. Here, we investigated whether injury to the cornea and/or changes in ECM composition (decorin knockout) can induce alterations within pachymeningeal tissue harboring, in addition to blood and lymphatic vessels, mast cells, resident macrophages, and mesenchymal stem cells, as such alterations can potentially lead to compromised microvascular integrity and local immune homeostasis thus creating the background for the development of neurodegenerative conditions.

## Methods

### Animals

In this study, C57BL/6J wild type (WT) from Jackson Laboratory (Bar Harbor, ME, USA) and decorin-deficient (Dcn^-/-^) mice, generated as previously described [12], were used. Animal procedures were approved by the Animal Care and Use Committees of the University of Missouri (Columbia, MO, USA) and the Harry S. Truman Memorial Veterans’ Hospital (Columbia, MO, USA). All experimental procedures were performed in adherence with the Association for Research in Vision and Ophthalmology (ARVO) statement for the use of animals in ophthalmic and vision research. In this study, female mice, seven to eight weeks of age were used. WT and DCN^-/-^ female mice were subjected to cornea alkali injury to investigate whether this injury could be associated with distant changes within cranial dura mater two weeks after initial insult. For this study, animals were divided into four groups: WT naive, 5 mice; WT with cornea injury (WT injury), 5 mice; Dcn^-/-^, 6 mice; and Dcn^-/-^ with cornea injury (DCN^-/-^ injury), 5 mice. The size of experimental groups was determined based on our previous experiences and Power Analysis. WT naïve animals, which did not have any genetic modifications and did not undergo any procedures, were used as control for all other groups.

### Corneal injury

Corneal injury was induced by a standard alkali burn injury method as described previously [13]. In brief, mice were anesthetized with an intraperitoneal injection of ketamine hydrochloride 100 mg/kg (JHP Pharmaceuticals, LLC, Rochester, MI, USA) and xylazine hydrochloride 10 mg/kg (XylaMed, Bimeda Inc., IL, USA). For topical anesthesia, a single drop of proparacaine hydrochloride 0.5% solution (Alcon, Ft. Worth, TX, USA) was instilled onto the eye. Corneal alkali injury was performed by placing a 2-mm diameter filter paper disc (P8, Filter Paper, Fisher Brand, Fisher Scientific, Pittsburgh, PA, USA), presoaked in Sodium hydroxide (NaOH) 0.5M solution (Sigma-Aldrich, St. Louis, MO, USA), onto the central cornea for 30 s, followed by extensive rinsing with balanced salt solution (Alcon, Fort Worth, TX) [10]. Alkali injury was performed in one eye only of each animal under the aid of a dissecting microscope (S6 E, Leica Microsystems Inc., Buffalo Grove, IL, USA).

### Tissue preparation, image acquisition, and analysis

**Fig 1:**
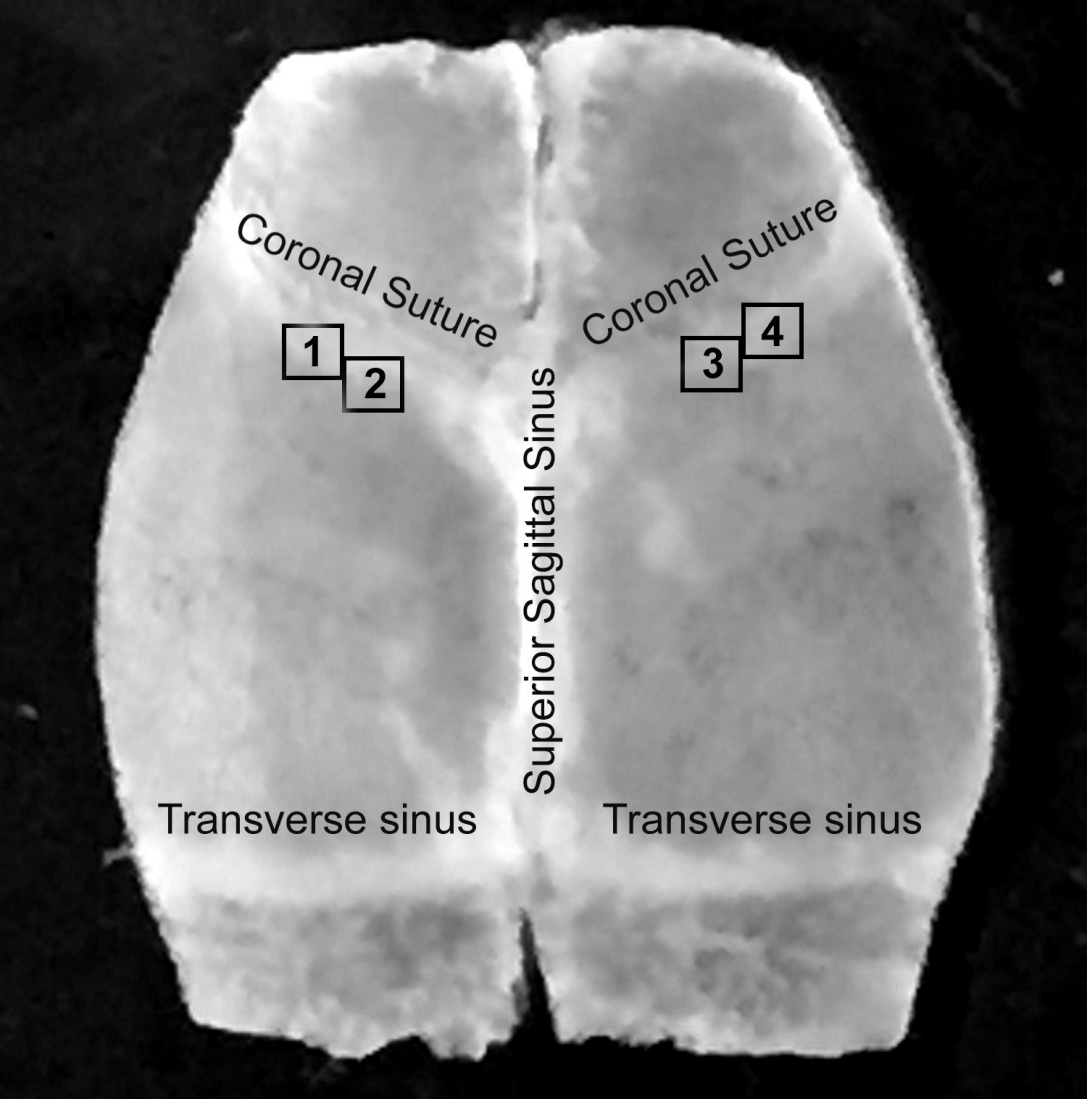
Anatomical locations of the investigated areas. Photographic image of the isolated mouse skull cap with cranial dura mater is shown. White squares labeled 1, 2, 3 and 4 in the left and right coronal suture regions designate investigated areas.

**Fig 2:**
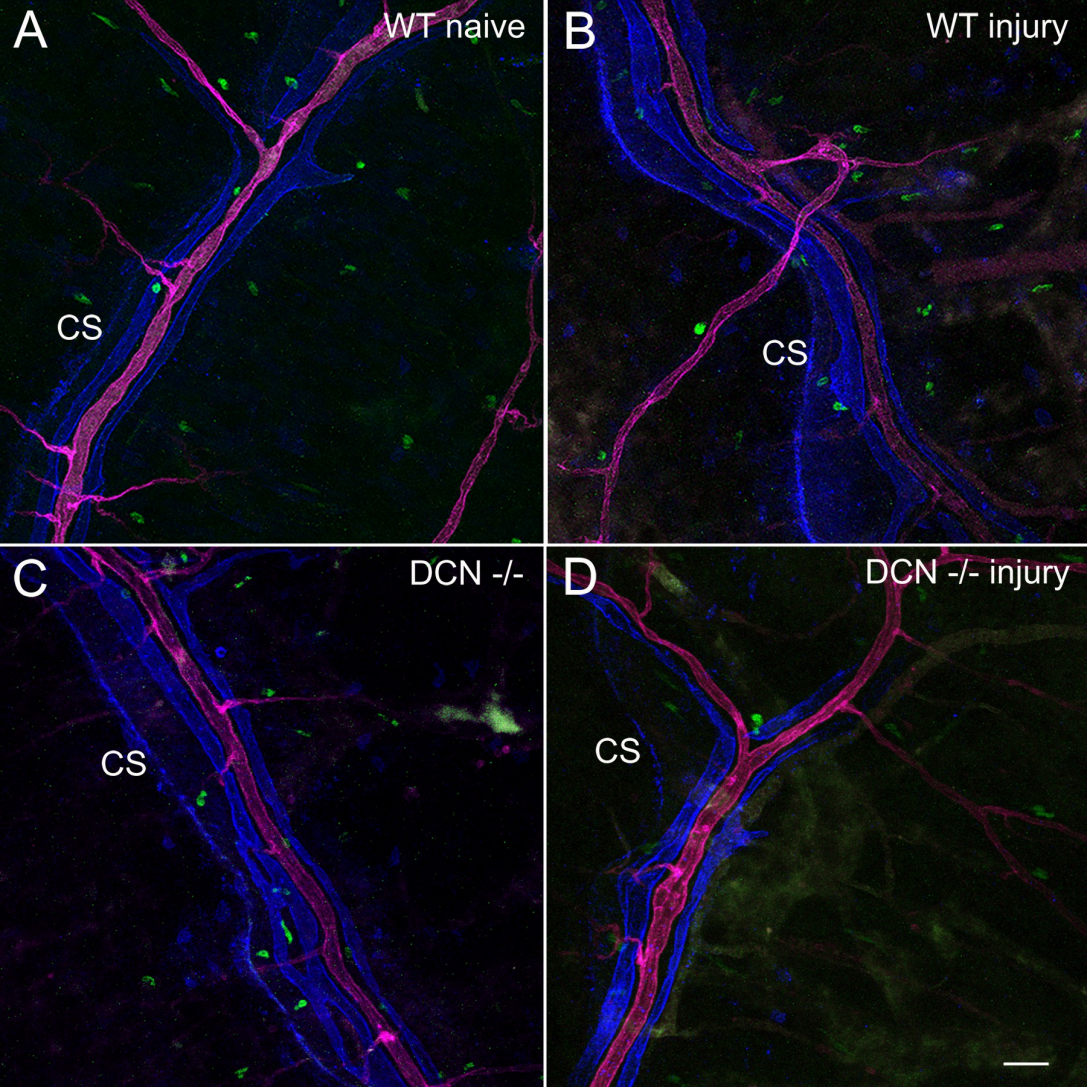
In the investigated area, PDPL and LYVE1 do not colocalize. Representative immunofluorescence images showing blood vessel staining with WGA lectin (magenta), PDPL-associated immunofluorescence (blue) and LYVE1 expression (green) in the CS investigation area taken with 20x objective. Note that in all 4 groups of animals LYVE1 expression (green) does not overlap with PDPL expression (blue). Scale bar in D, 50 μm.

Two weeks after corneal injury mice were sacrificed with CO2 inhalation. Immediately following sacrifice, the chest cavity was opened, and the entire body of the mouse was perfused through the heart with 1 ml of Kreb’s/BSA containing 20 μg/ml AlexaFluor 594-conjugated wheat germ agglutinin (WGA) lectin (Thermo Fisher Scientific, Berkeley, MO, USA, Cat # W11262) and AlexaFluor 488-conjugated anti-mouse LYVE1 (lymphatic vessel endothelial hyaluronan receptor 1) antibody (Thermo Fisher Scientific, Berkeley, MO, USA, clone ALY7 rat IgG1 kappa, Cat # 53-0443-82, 1:100 final dilution) to identify blood microvessels and lymphatic structures respectively [14]. Dorsal cranium with the dura mater was removed and fixed in 4% paraformaldehyde. After 24 h tissue sample was washed with PBS and permeabilized with 0.5% Triton X-100 overnight. Non-specific sites were masked with blocking reagent (3% BSA in PBS) and tissue samples were subjected to two immunostaining setups as follows. The first setup of immunostaining was carried out using primary anti-podoplanin (PDPL) antibody (Abcam, Boston, MA, USA, Cat # ab11936, 10 μg/ml, overnight incubation at 4°C) followed by the secondary Alexa Fluor® 647-conjugated goat anti-Syrian hamster IgG (Abcam, Boston, MA, USA, ab180117, 5 μg/ml) for 2 hours at room temperature. After washing in PBS for 5 min three times and mounting on a slide using ibidi Mounting Medium for fluorescence microscopy (ibidi Inc., Fitchburg, WI, USA, Cat # 50001), images at 512x512 pixels were taken using Fluoview FV1000 confocal microscope (Olympus) with software FV10-ASW at defined (around middle 1/3) anatomical locations in the coronal suture (CS) area (Fig 1). This area was chosen because CS provides a necessary anatomical reference hallmark enabling for investigating and comparing exactly the same DM regions in all animals. Also, in these areas, we recently discovered advanced microvascular lymphatic networks, which were never described before. unlike the area close to the bregma (point of conjunction of CS and Superior Sagittal Sinus), in these DM regions appreciable blood and lymphatic microvascular networks consistently could be detected. Z-stacks (180–200 images) were acquired using 20x objective with 1μm step size. For the second immunostaining setup, tissue samples were unmounted, washed in PBS for 5 min 5 times and stained with FITC-conjugated anti-α-smooth muscle actin (α-SMA) antibody (Sigma-Aldrich, St. Louis, MO, USA, Cat # F3777-.2ML, 3 μg/ml) and anti-fibroblast specific protein 1 (FSP1) antibody (Thermo Fisher Scientific, Cat # PA5-95736, 15 μg/ml) followed by the secondary Alexa Fluor® 594-conjugated goat anti-rabbit IgG (Thermo Fisher Scientific, Cat # A11072, 5 μg/ml). Samples were remounted and images were taken using the same imaging system with the same settings at exactly the same anatomical locations within the coronal suture area as above (Fig 1). Thus, two sets of images from each exact location were acquired. Using National Institutes of Health ImageJ2 (NIH, Bethesda, MD) software (2.3.0/1.53q), images generated with the first immunostaining setup were subtracted from images of the identical locations generated with second immunostaining setup to eliminate any possible overlapping of fluorescent signals. Image histograms generated in ImageJ were employed for quantitative analysis of PDPL, LYVE1, and WGA-associated fluorescence from the first immunostaining setup, and FSP1 and α-SMA-associated fluorescence from the second immunostaining setup images. Images from all four experimental groups of animals were analyzed with particular emphasis on dura connective tissue stroma and blood vessel wall integrity at defined anatomical locations (Fig 1). Our analysis was focused on distribution and mean fluorescence intensity of five markers that allow to identify different vascular structures within dura mater as well as elucidate morphological changes within the vasculature and tissue stroma following two weeks after corneal injury. WGA staining was used to identify meningeal blood vessels, while PDPL and LYVE1 immunoreactivity to identify dural lymphatics and nonvascular LYVE1-positive immune cells. Interestingly, in this area, lymphatic vessels do not actually express LYVE1 (Fig 2). Its expression is limited to nonvascular cells parts of tissue stroma, and blood vessel perivascular expression. Consequently, LYVE1 expression was used to analyze only overall mean fluorescence intensity and calculate the number of nonvascular LYVE1 positive cells. On the other hand, lymphatic vessels here are characterized by PDPL expression. Accordingly, PDPL fluorescence was used to determine the number of PDPL^+^ lymphatic sprouts and PDPL^+^ lymphatic vessel (LV) index. PDPL-positive lymphatic sprouts were counted by two independent observers in all photomicrographs followed by discussing and reaching a consensus on all sprouts in question. LV index was determined by creating a point array graticule using the Grid tool of ImageJ2 software and calculating the number of grid points that hit PDPL^+^ vessels. FSP1 and α-SMA positivity were used as the markers of activated fibroblasts. This choice was based on several studies that confirmed FSP1 and α-SMA as markers for post-injury activated fibroblasts [15–19] showing that they (FSP1 and α-SMA) are absent in the quiescent fibroblasts. In addition, α-SMA immunostaining also allowed to identify vascular smooth muscle cells (VSMCs). To quantitate changes in VSMC coverage of meningeal blood vessels, first deep learning cascades were used for blood vessel segmentation as previously described [20] to determine blood vessel boundaries and calculate blood vessel network area in each microscopic image. Next, for each image, α-SMA associated immunofluorescence signal was measured over the blood vessel network area, and the ratio of α-SMA associated immunofluorescence to blood vessel network area calculated to reflect blood vessel VSMC coverage.

### Statistical analysis

One-way analysis of variance (ANOVA) with Tukey post hoc test were used for statistical analysis. The data were expressed as fold change in fluorescence intensity compared with control. GraphPad Prism 9 (GraphPad Software, La Jolla, CA) software was used for statistical analysis and p ≤ 0.05 was used as the level of significance. See S1 File for a detailed breakdown of FSP1 and LYVE1 fluorescence intensity data as well as PDPL^+^ sprout and LYVE1^+^ cell counts in each individual sample.

## Results

### **Alkaline corneal injury does not induce changes in the overall WGA lectin- and α**-**SMA-associated fluorescence in the CS area of mouse dura mater**

In our experiments, two weeks post corneal injury, we did not detect statistically significant changes in WGA lectin- or α-SMA-associated fluorescence in the investigated dura mater areas in either WT or DCN^-/-^ mice. As WGA lectin is used to detect blood vessel endothelial cells and α-SMA to detect vascular smooth muscle cells, this implies that no major changes in the overall vascular density of the investigated dura regions occurred within two weeks of the initial insult.

### Corneal injury triggers meningeal remodeling associated with tissue stroma alterations and morphological changes within dura mater VSMCs compromising blood vessel integrity

**Fig 3:**
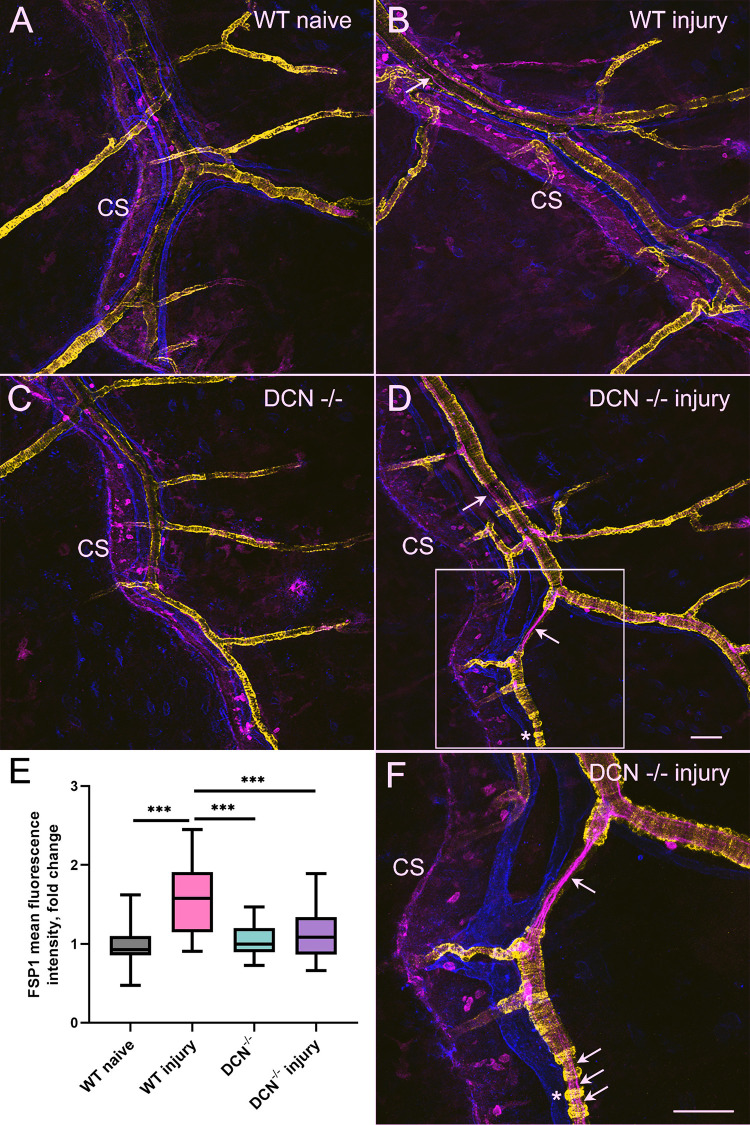
Dura mater stromal and vascular responses to alkaline corneal injury. Representative immunofluorescence images of FSP1 (magenta), PDPL (blue) and α-SMA (yellow) expression from investigation area 3 taken with 20x objective. Note significantly elevated stromal FSP1 expression in WT injury group (B) compared with WT naïve (A), DCN^-/-^ (C) and DCN^-/-^ injury (D) animals. Also note endothelial (white arrows in B, D and F) FSP1 expression in WT injury (B) and DCN^-/-^ injury mice two weeks post initial insult. In F, area marked with the white square in D was imaged using 40x objective to show in more detail post injury endothelial FSP1 expression (white arrows), as well the loss of VSMC coverage of the blood vessels and acquisition of bead-like morphology by VSMCs (white asterisk). E, box diagram depicting fold change of in FSP1-associated immunofluorescence in four experimental groups compared with WT naïve animals. Whiskers, minimal to maximal, *P*–Ordinary One-Way ANOVA, *** P < 0.0001. Scale bars in D and F, 50 μm. For black and white images depicting each channel individually for FSP1, PDPL, and α-SMA please see S1–S3 Figs correspondingly.

**Fig 4:**
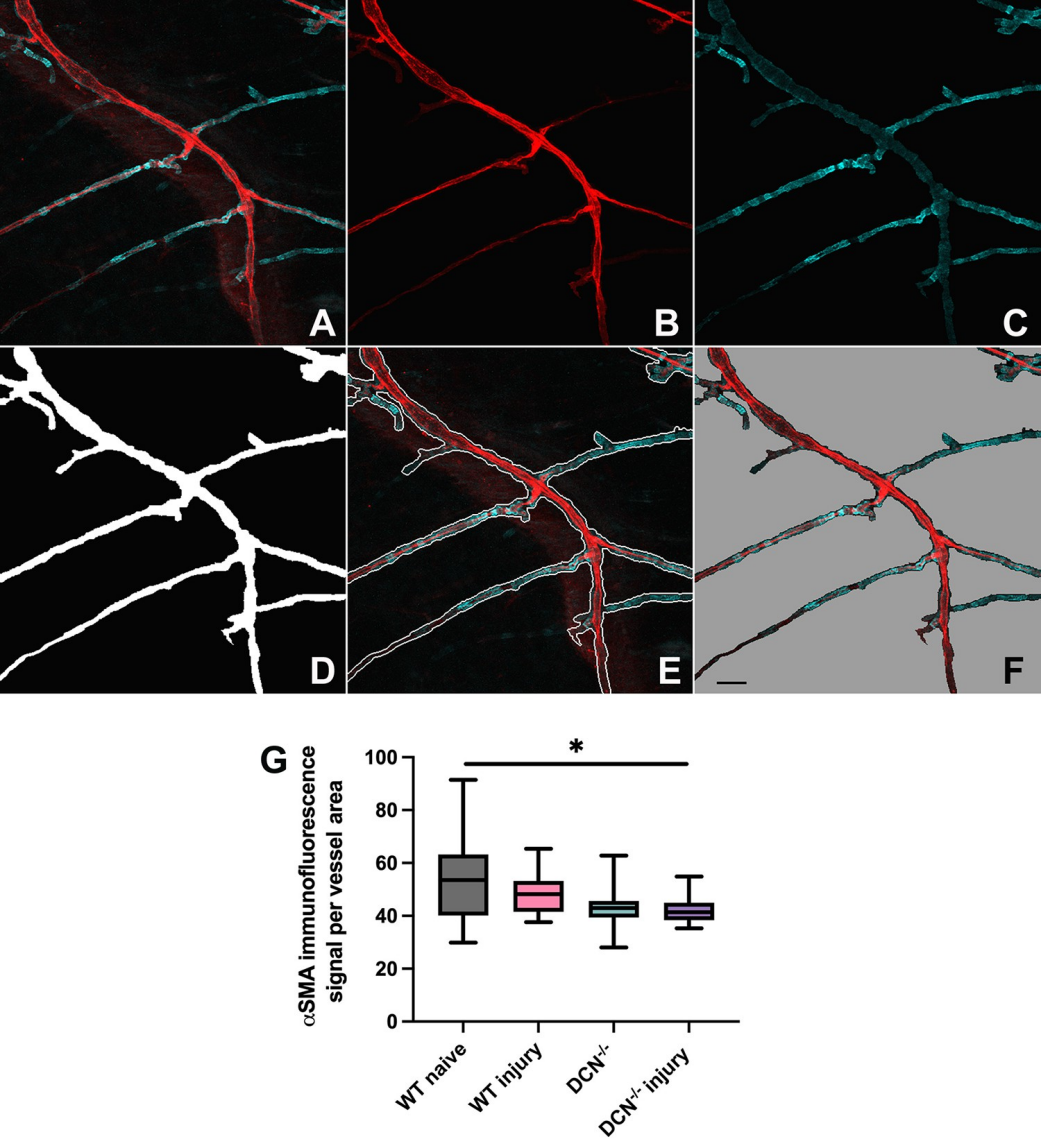
Changes in blood vessel VSMC coverage two weeks post alkaline corneal injury. A through F, blood vessel segmentation stages are depicted. In A through F, red channel represents blood vessel wall WGA lectin staining and blue channel represents α-SMA VSMC staining. The ensemble of deep learning cascades was used to perform blood vessel segmentation to enable quantitation of VSMC coverage. In A, the original RGB image is shown. In B and C, masked red and blue channels are shown respectively. In D. vessel mask is shown whilst in E, vessel tree boundary is applied over the original image. Blood vessel region on the original image is shown in F with gray color marking the background. G, box diagram depicting fold change in α-SMA-associated immunofluorescence over blood vessel area in four experimental groups compared with WT naïve animals. Whiskers, minimal to maximal, *P*–Ordinary One-Way ANOVA, * P < 0.05. Scale bar in F, 50 μm.

In this study, we used FSP1, which is often utilized as a marker of fibroblast activation [15–19], to assess the potential for connective tissue remodeling in mouse dura mater induced by alkaline corneal injury. Our data demonstrate enhanced FSP1 immunoreactivity in dura mater of WT animals two weeks post corneal injury compared with WT naïve animals (Fig 3A, 3B and 3E). Remarkably, such reaction was not observed in DCN^-/-^ mice (Fig 3C–3E). These results suggest that injury of the cornea can activate dural fibroblasts, which could potentially facilitate the development of the fibrotic process within meningeal stroma two weeks after initial insult. However, in DCN^-/-^ mice, decorin deficiency modifies such connective tissue response to the injury (Fig 3A–3E). Interestingly, two weeks after corneal injury in WT mice, FSP1 immunoreactivity has been also detected within blood vessel walls (Fig 3B white arrow), showing a similarity with the population of endothelial cells expressing FSP1, which has been described in the injured mouse heart [19,21]. These vascular wall alterations two weeks following initial insult (endothelial FSP1 expression) are even more evident in DCN^-/-^ mice (Fig 3D and 3F), suggesting that deficiency of the major ECM component, small leucine-rich proteoglycan decorin, modifies the late response to the injury within blood vessel walls. Interestingly, in DCN^-/-^ mice, the enhanced post corneal injury FSP1 immunoreactivity within meningeal blood vessel walls is paralleled by morphological changes in VSMC and impaired mural cell blood vessel coverage (Fig 3D and 3F white arrows and Fig 4). A dropout of smooth muscle cells reducing their vessel wall coverage has been detected on blood vessels of different calibers in DCN^-/-^ animals following the injury (Figs 3D, 3F and 4), strongly suggesting major impairment of dura mater blood vessels integrity in DCN^-/-^ mice.

### Morphological changes within PDPL-positive lymphatic vasculature two weeks after corneal injury

**Fig 5:**
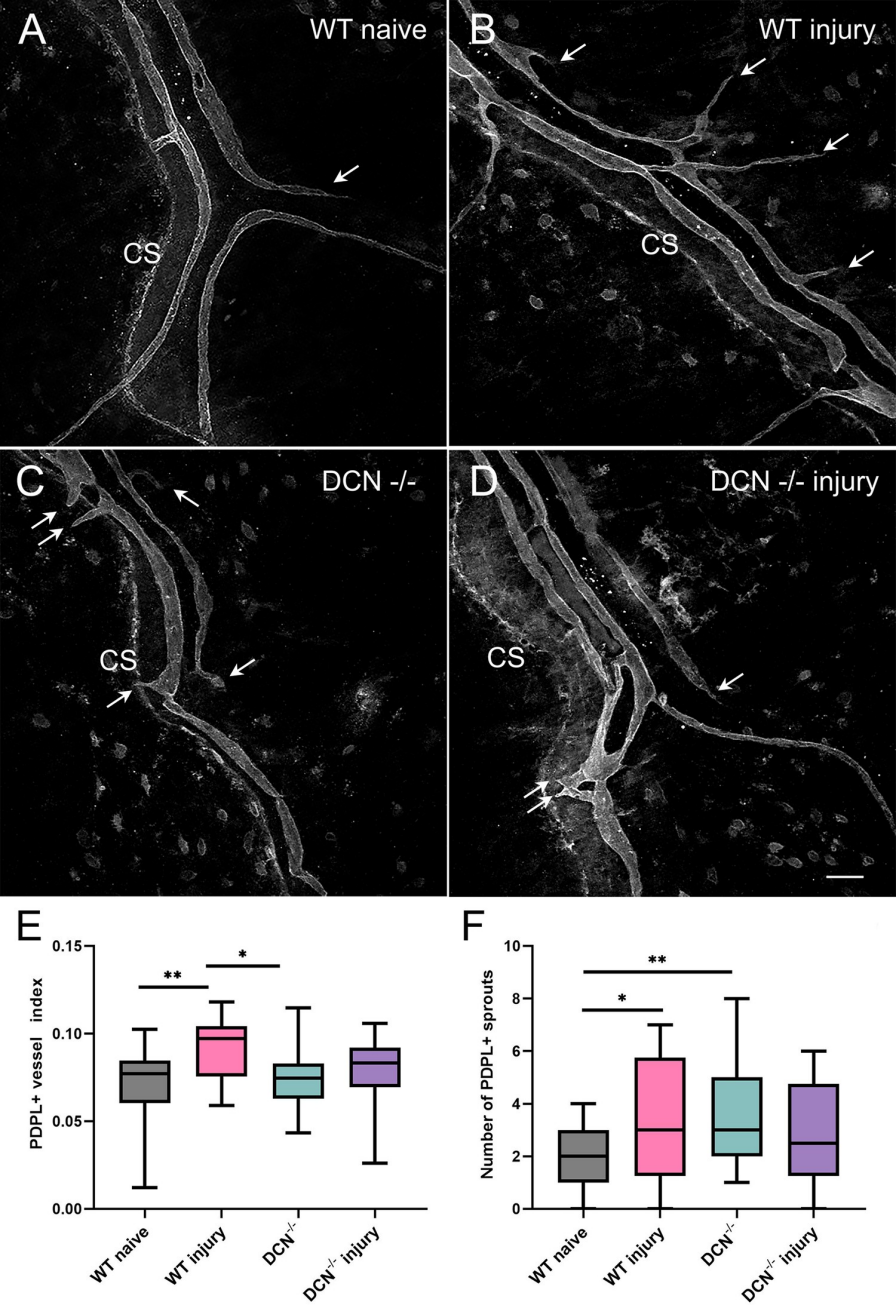
Changes in the number of PDPL positive dura mater lymphatic sprouts two weeks post alkaline corneal injury. PDPL^+^ meningeal lymphatics were visualized using primary anti-PDPL antibody followed by the secondary Alexa Fluor® 647-conjugated goat anti-Syrian hamster IgG. Note significant increase in PDPL^+^ lymphatic vessel index in WT injury (B) group compared with WT naïve animals (A), as well as the number of PDPL^+^ lymphatic sprouts (white arrows) in WT injury (B) and DCN^-/-^ (C) groups, but not in DCN^-/-^ injury (D) mice compared with WT naïve (A) mice. E and F, box diagrams showing changes in PDPL^+^ dura mater lymphatic vessel index and the number of PDPL^+^ sprouts two weeks post alkaline corneal injury. Whiskers, minimal to maximal, *P*–Ordinary One-Way ANOVA, * *P* < 0.05; *** *P* < 0.0001. Scale bar in D, 50 μm.

Dura mater lymphatic system was identified as a critical mediator of CNS drainage [22]. Thus, we analyzed whether PDPL-positive meningeal lymphatics are impacted by corneal alkaline injury. Within the investigated areas, PDPL-positive immunoreactivity is associated with PDPL-expressing lymphatic vessels, individual cells and select regions of the CS itself (Fig 5). As analyzed by mean fluorescence intensity or the percent of area coverage by PDPL-positive structures, there was no statistically significant difference between the four experimental groups. However, our results reveal that morphological changes within PDPL-positive lymphatic vessels two weeks post corneal injury do occur as manifested by the significant increase in the number of PDPL^+^ vessel sprouts. (Fig 5). Quantitative analysis of the number of PDPL^+^ vascular sprouts within investigated areas demonstrate a significant increase in their number after corneal injury in WT injury group compared to WT naïve mice (Fig 5A, 5B and 5E). This is reflective of the increase in lymphatic vasculature complexity and could be indicative of the lymphangiogenic process within cranial meninges as a delayed response to corneal injury. Interestingly, the ECM modification (lack of decorin) in DCN^-/-^ mice caused increase in the number of PDPL^+^ sprouts compared with WT naïve animals similar to WT injury group. However, the lymphangiogenic responses in DCN^-/-^ injury mice were blunted and the number of PDPL^+^ sprouts was not significantly different from either WT naïve group, or DCN^-/-^ mice.

### Altered LYVE1 expression within cranial dura mater is not associated with corneal injury in WT mice, but is evident in DCN^-/-^ animals

**Fig 6:**
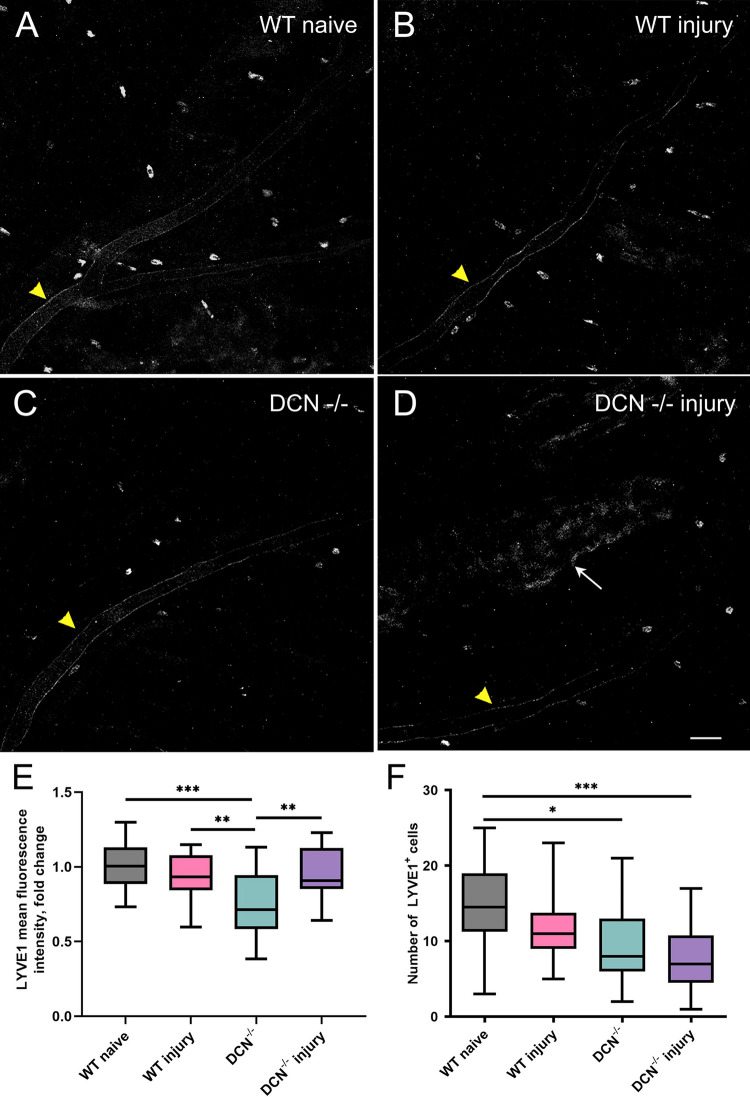
Altered LYVE1 expression within CS area of the cranial dura mater in DCN^-/-^ animals. The expression of lymphatic vessel endothelial hyaluronan receptor LYVE1 (green) was visualized using AlexaFluor 488-conjugated anti-mouse LYVE1 antibody. Note that in addition to nonvascular stromal LYVE1 expression and individual nonvascular LYVE1^+^ cells, perivascular blood vessel LYVE1 expression (yellow arrowheads in A through D) could be observed. However, there was no LIVE1 positive lymphatic vessels found in DM areas analyzed in this study. Compared with WT naïve animals (A) overall LYVE1 associated immunofluorescence did not change significantly in WT injury group (B). However, compared with WT naïve and WT injury animals, it was significantly reduced in DCN^-/-^ mice (C). Interestingly, two weeks post alkaline corneal injury, dura mater nonvascular tissue stroma LYVE1 expression increased significantly in DCN^-/-^ injury group (D, white arrow). In addition to the overall LYVE1 associated immunofluorescence, the number of nonvascular LYVE1 positive cells was also analyzed. Compared with WT naïve animals, the number of nonvascular LYVE1^+^ cells did not change significantly in WT injury group. However, it was significantly reduced in DCN^-/-^ and DCN^-/-^ injury mice. E and F, box diagrams showing fold change differences in the overall LYVE1 associated immunofluorescence compared with WT naïve animals (E) and the number of nonvascular LYVE1^+^ cells (F). Whiskers, minimal to maximal, *P*–Ordinary One-Way ANOVA, * *P* < 0.05; ** *P* < 0.01; *** *P* < 0.0001. Scale bar in D, 50 μm.

In the current study we assessed LYVE1 immunoreactivity on cranial dura mater samples, which include vascular/tissue staining and nonvascular LYVE1-positive populations of immune cells. As described in the literature, these nonvascular LYVE1-positive cells are mostly CD68, CD163, and CD206 expressing macrophages [23–26], bone marrow-derived mesenchymal stem cells [27], CD45^+^, CD11b^+^ bone marrow-derived monocytic lineage cells [28], and dendritic cells [29]. Thus, in addition to quantitating overall LYVE1-associated fluorescence intensity of the samples (Fig 6E), the LYVE1^+^ cell count was assessed as a separate factor (Fig 6F). Our data show that, whilst LYVE1 mean fluorescence intensity in WT injury group did not differ significantly from WT naïve mice, it was significantly decreased in DCN^-/-^ animals compared with both the WT naïve and WT injury groups (Fig 6). However, in DCN^-/-^ injury mice, the overall LYVE1 fluorescence did increase significantly compared with DCN^-/-^ animals (Fig 6). Interestingly, it appears that this increase is mostly associated with elevated vascular/tissue LYVE1 expression [30], as the number of LYVE1+ cells in both DCN^-/-^ and DCN^-/-^ injury groups was significantly lower than in WT naïve mice (Fig 6). These results suggest that ECM modification by DCN deficiency can alter LYVE1 expression on vascular/tissue structures and population density of nonvascular immune cells leading to a reduction in the number of meningeal LYVE1-positive immune cells. This is consistent with recent literature data suggesting that LYVE1 may contribute more to immune surveillance than fluid homeostasis [31–33].

## Discussion

In this study, we investigated select aspects of the late (two weeks post initial insult) responses of fibrous dura mater connective tissue and meningeal vascular system to alkaline corneal injury in WT and DCN^-/-^ animals, as well as how changes in ECM composition (DCN deficiency) modify these responses.

It has been shown that alkaline corneal injury can stimulate inflammation propagating to the trigeminal ganglion (TG) through the cornea-trigeminal axis [34]. This early inflammatory process is mediated by sensory neurotransmitters released from the trigeminal neurons [35] also supplying sensory innervation to both vascular and stromal dural territories [36,37]. We speculate that early dural responses to corneal injury, which require separate investigation, most likely involve a cascade of events within highly innervated and vascularized dura mater connective tissue and depends on the local distribution of meningeal perivascular or stromal nociceptors [38]. It has been reported that CGRP (calcitonin gene-related peptide) or SP (substance P) neuropeptide release from activated nociceptors is associated with vasodilation, plasma protein leakage, extravasation of leukocytes, tissue swelling, mast cell activation [39] and augmented mechanosensitivity [40] leading to the impaired meningeal vascular integrity. All these major determinants of the outcomes from early responses can contribute to the late or chronic post corneal injury events within the dura tissue.

Our data indicate that two weeks after the initial alkaline corneal injury, a marked increase in FSP1 stromal expression is detected (Fig 3A, 3B and 3E), which is indicative of fibroblast activation and potential pro-fibrotic changes in CS area of dura mater stroma in WT mice. Under normal conditions fibroblast-like cells, expressing FSP1 are found in perivascular locations [41]. They were also found to be interposed between blood vessels and dural macrophages, which could be aligned along blood vessels or located in DM parenchyma [42]. However, after injury FSP1 expression can be induced in endothelial cells as has been shown in injured mouse heart model [19,21]. Indeed, similarly to injured cardiac endothelial cells [19], FSP1 endothelial expression was detected in our experiments in both WT and DCN-/- mice (Fig 3B, 3D and 3F white arrows). However, modifying ECM composition by knocking out DCN blunted stromal FSP1 responses in DCN^-/-^ animals (Fig 3).

ECM changes could also lead to transdifferentiation of SMC or endothelial (through endothelial-mesenchymal transition) cells [43] to myofibroblasts [44]. Remarkably, in our experiments, two weeks post injury DCN^-/-^ mice exhibited more pronounced vascular wall alterations involving changes in VSMC morphology (which could potentially lead to the impaired vascular integrity) than WT animals (Fig 3).

Vascular stability and integrity are maintained at different levels that include endothelial cells, mural cells, and surrounding stroma including ECM [45]. The ECM is a significant determinant of microvascular structure and function. It’s composition, stiffness and fiber orientation have a regulatory influence on vascular cells [46]. The ECM composition and structure are dynamically altered during various physiological and pathological conditions and modulate vascular remodeling [47]. Deficiencies or mutations in ECM proteins causing vasculature-related pathological phenotypes have been identified [46]. For example, biglycan deficiency/mutation cause spontaneous aortic dissection and rupture in mice [48] and thoracic aortic aneurysms and dissections in humans [49]. The other small leucine-rich proteoglycan, DCN, which regulates cell proliferation, survival, and differentiation [50], has been extensively studied in eye research [51–53]. Tissue specific viral gene transfer of DCN reduces corneal scarring [54] through its inhibitory effect on TGF-β in corneal fibroblasts [55]. It appears that DCN is playing an important role in regulating dura mater stromal and vascular responses to corneal injury. DCN, besides being a structural ECM component, has multifaceted functions acting as a signaling molecule [30]. In our experiments, DCN deficiency modifies responses to corneal injury within meningeal stroma and blood vessel walls (Fig 3). In DCN^-/-^ mice FSP1 responses within dura stroma two weeks following cornea injury were blunted compared with WT mice. However, morphological changes within blood vessel walls were more evident in DCN^-/-^ animals (Fig 3). α-SMA staining revealed structural alterations within blood vessel walls, including changes in VSMC morphology and reduced VSMC coverage of blood vessels of different calibers that were most pronounced in DCN^-/-^ injury group (Figs 3 and 4). Thus, in addition to regulating the dynamic VSMC-ECM crosstalk [56], DCN is also one of the important players for preserving meningeal vascular integrity. Indeed, DCN deficiency induces dysregulated TGFβ signaling as a result of the impaired DCN-mediated TGFβ sequestration and ultimately leads to TGFβ overproduction. It has been shown that chronic TGFβ overproduction causes a dropout of smooth muscle cells and pericytes reducing their coverage on cerebral blood vessels [57] similar to what we observe in DCN^-/-^ injury group (Figs 3 and 4). Reduced VSMC vessel coverage along with VSMCs acquisition of beads-like morphology (Fig 3) indicate possible changes in VSMC phenotype related to the remodeling of the vascular wall, which frequently play role in vascular diseases. While deficiencies or mutations in ECM proteins have been shown previously to cause VSMCs phenotype switching [47], this study demonstrates for the first time that delayed (two weeks after initial insult) meningeal responses to corneal injury are associated with the VSMC morphological alterations associated with impaired meningeal blood microvessel integrity, which could be clinically relevant.

With respect to the meningeal lymphatics, our data reveal that alkaline corneal injury is associated with morphological alteration within PDPL-positive meningeal lymphatic vessels. As a response to the corneal injury, two weeks after the initial insult, we detected an increase in PDPL^+^ lymphatic vessel index and the number of PDPL^+^ vascular sprouts in WT mice dura mater (Fig 5). A rising number of PDPL^+^ sprouts suggests an increase in overall meningeal lymphatic vasculature complexity and indicates lymphangiogenic response [58]. Interestingly, ECM modification in DCN^-/-^ mice leads to the similar increase in the number of PDPL^+^ sprouts compared with WT naïve animals, however, with no further increase in DCN-/- injury experimental group.

Our results suggest that DCN also plays an important role in regulating tissue/vascular LYVE1 expression and controlling population of dura mater nonvascular LYVE1^+^ cells (Fig 6), which include immune cells such as macrophages, lymphocytes, dendritic cells, innate lymphoid cells, and T cells [23–29]. These LYVE1+ cells may act as a reservoir cell recruitment when the immune system is challenged [28]. Within cranial dura, physical proximity of sensory neurons and immune cells [36,59,60] not only allows for them to interact directly with each other, but also to share a common microenvironmental niche (including ECM) for surveying tissue integrity [60]. Consequently, ECM composition (DCN expression) could be an important factor regulating immune surveillance within cranial dura mater.

In summary, this study demonstrates for the first time that two weeks post initial insult alkaline corneal injury induces pathological processes in CS area of mouse dura mater manifested by significant pro-fibrotic changes in dural stroma, vascular remodeling characterized by alterations in VSMC morphology, reduced blood vessel wall VSMC coverage and endothelial cell FSP1 expression, as well as significant increase in the number of PDPL^+^ sprouts. At this time, we do not know whether these changes are DM specific, or other tissues including the brain could be affected as well. Furthermore, even within dura, these responses could be region-specific. Interestingly, even though only one eye (left) was challenged by the alkaline injury, the stromal and vascular changes in DM were not ipsilateral and two weeks post injury were detected on both sides. Mechanistically, the described dural responses to corneal injury depend on ECM composition (DCN expression). While these results may have important clinical implications, the study itself has some limitations. Only female animals were used, so whether there are any sex differences in these responses could not be established. Also, only one time point (2 weeks post injury) was investigated. Further longitudinal studies will be performed to investigate more immediate dural responses to corneal injury as well as long term consequences to find out whether these changes are permanent or resolve over time similar to changes in DM lymphatics following traumatic brain injury [22]. Also, it would be important to unveil finite mechanisms of the transmission of pathological stimuli from cornea to meninges.

## Supporting information

S1 Fig Dura mater stromal and vascular responses to alkaline corneal injury. Representative immunofluorescence images of FSP1 expression from investigation area 3 taken with 20x objective. Note significantly elevated stromal FSP1 expression in WT injury group (B) compared with WT naïve (A), DCN-/- (C) and DCN-/- injury (D) animals. Also note endothelial (white arrows in B, D and F) FSP1 expression in WT injury (B) and DCN-/- injury mice two weeks post initial insult. Area marked with the white square in D was imaged using 40x objective (see Fig 2, F in the manuscript) to show in more detail post injury endothelial FSP1 expression (white arrows). Scale bar in D, 50 μm.
(PDF)

S2 Fig Dura mater stromal and vascular responses to alkaline corneal injury. Representative immunofluorescence images of PDPL expression from investigation area 3 taken with 20x objective. While there was no statistically significant difference between the groups in the overall PDPL expression, there was a significant increase in PDPL+ lymphatic vessel index in WT injury (B) group compared with WT naïve animals (A), as well as the number of PDPL+ lymphatic sprouts in WT injury (B) and DCN-/- (C) groups, but not in DCN-/- injury (D) mice compared with WT naïve (A) mice (see Fig 3 in the manuscript for details). Scale bar in D, 50 μm.
(PDF)

S3 Fig Dura mater stromal and vascular responses to alkaline corneal injury. Representative immunofluorescence images of α-SMA expression from investigation area 3 taken with 20x objective. Note the loss of VSMC coverage of the blood vessels (white arrows) and acquisition of bead-like morphology by VSMCs (white asterisk). Area marked with the white square in D was imaged using 40x objective (see Fig 2, F in the manuscript) to show in more detail post injury changes in blood vessel VSMC coverage and VSMC morphology. Scale bar in D, 50 μm.
(PDF)

S1 File FSP1, LYVE1 fluorescence intensity, PDPL+ sprout, LYVE1+ cell counts. (XLSX)
