# Supplementary material for: Corneal injury is associated with stromal and vascular alterations within cranial dura mater
Source: PLoS One. 2023 Apr 20;18(4):e0284082. doi: 10.1371/journal.pone.0284082 (PMC10118146; doi:10.1371/journal.pone.0284082)
Supplement: S1 Fig — Representative immunofluorescence images of FSP1 expression from investigation area 3 taken with 20x objective. Note significantly elevated stromal FSP1 expression in WT injury group (B) compared with WT naïve (A), DCN-/- (C) and DCN-/- injury (D) animals. Also note endothelial (white arrows in B, D and F) FSP1 expression in WT injury (B) and DCN-/- injury mice two weeks post initial insult. Area marked with the white square in D was imaged using 40x objective (see Fig 2, F in the manuscript) to show in more detail post injury endothelial FSP1 expression (white arrows). Scale bar in D, 50 μm. (PDF) [file pone.0284082.s001.pdf]

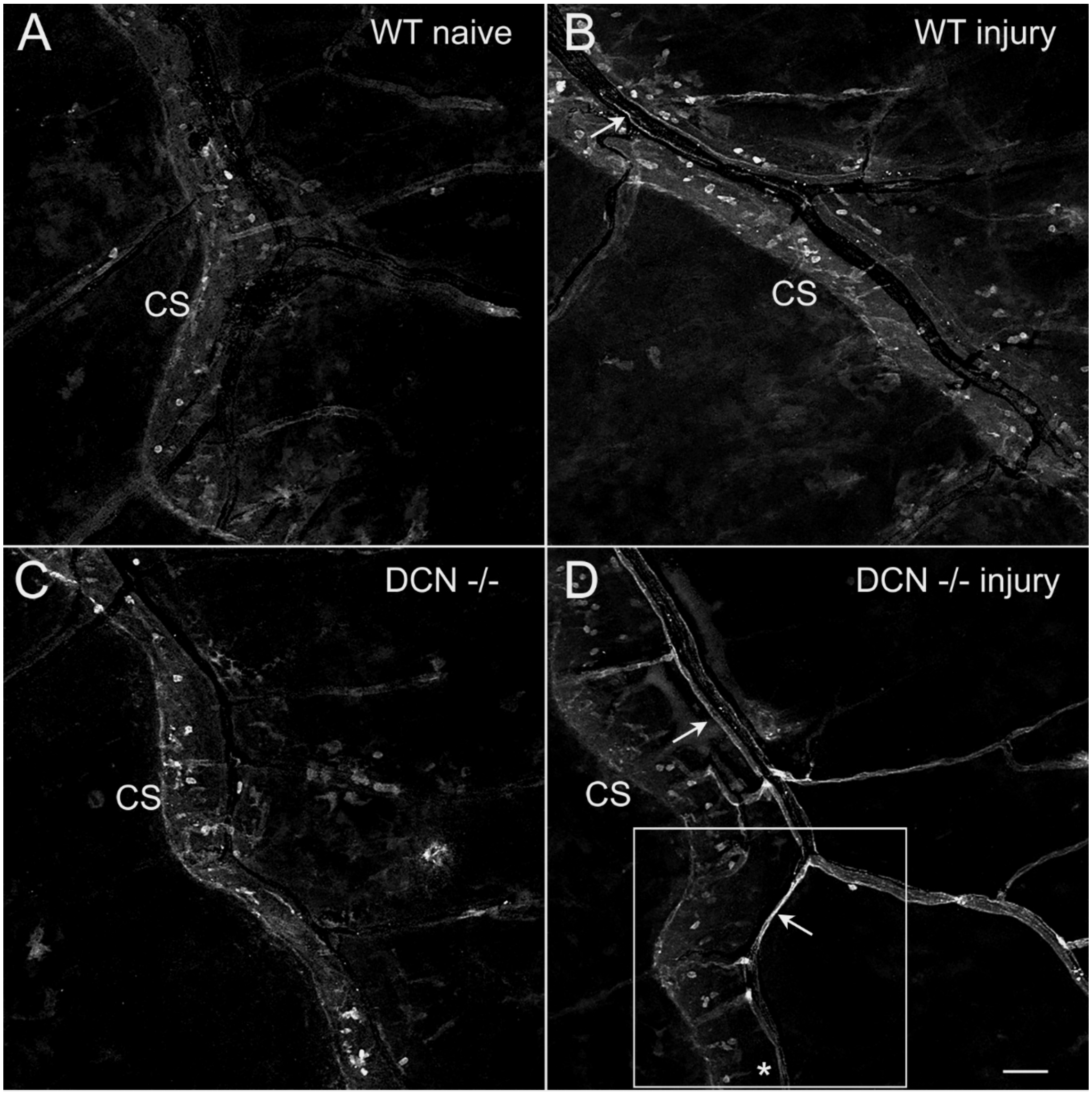

Supplemental Figure S1. Dura mater stromal and vascular responses to alkaline corneal injury. Representative immunofluorescence images of FSP1 expression from investigation area 3 taken with 20x objective. Note significantly elevated stromal FSP1 expression in WT injury group (B) compared with WT naïve (A), $DCN^{-/-}$ (C) and $DCN^{-/-}$ injury (D) animals. Also note endothelial (white arrows in B, D and F) FSP1 expression in WT injury (B) and $DCN^{-/-}$ injury mice two weeks post initial insult. Area marked with the white square in D was imaged using 40x objective (see Figure 2, F in the manuscript) to show in more detail post injury endothelial FSP1 expression (white arrows). Scale bar in D, 50 μm.
