# Supplementary figures and images for: Corneal injury is associated with stromal and vascular alterations within cranial dura mater
Source: PLoS One. 2023 Apr 20;18(4):e0284082. doi: 10.1371/journal.pone.0284082 (PMC10118146; doi:10.1371/journal.pone.0284082)

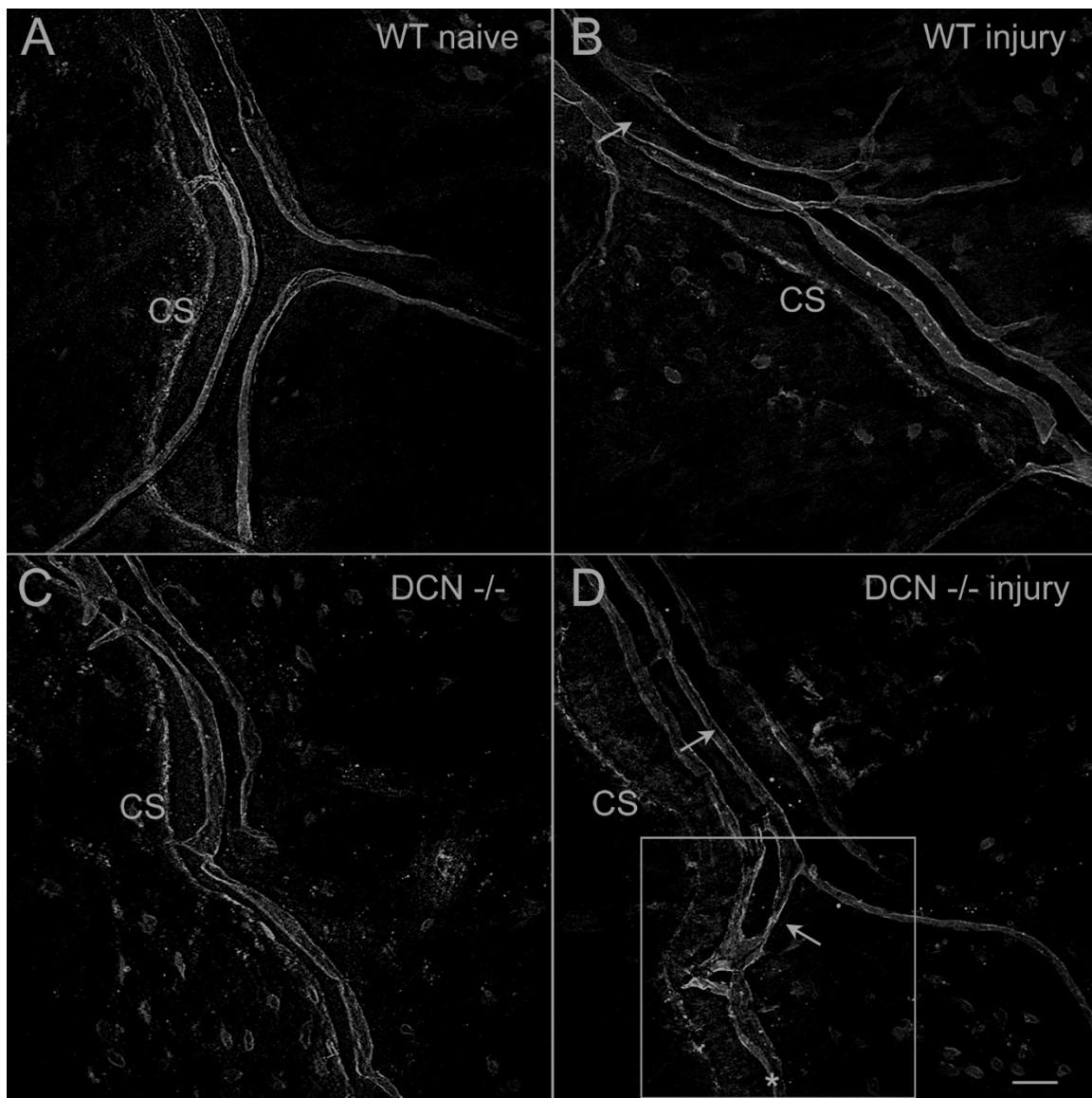

Supplement: S2 Fig — Representative immunofluorescence images of PDPL expression from investigation area 3 taken with 20x objective. While there was no statistically significant difference between the groups in the overall PDPL expression, there was a significant increase in PDPL+ lymphatic vessel index in WT injury (B) group compared with WT naïve animals (A), as well as the number of PDPL+ lymphatic sprouts in WT injury (B) and DCN-/- (C) groups, but not in DCN-/- injury (D) mice compared with WT naïve (A) mice (see Fig 3 in the manuscript for details). Scale bar in D, 50 μm. (PDF) [file pone.0284082.s002.pdf]
