# Supplementary material for: Corneal injury is associated with stromal and vascular alterations within cranial dura mater
Source: PLoS One. 2023 Apr 20;18(4):e0284082. doi: 10.1371/journal.pone.0284082 (PMC10118146; doi:10.1371/journal.pone.0284082)
Supplement: S3 Fig — Representative immunofluorescence images of α-SMA expression from investigation area 3 taken with 20x objective. Note the loss of VSMC coverage of the blood vessels (white arrows) and acquisition of bead-like morphology by VSMCs (white asterisk). Area marked with the white square in D was imaged using 40x objective (see Fig 2, F in the manuscript) to show in more detail post injury changes in blood vessel VSMC coverage and VSMC morphology. Scale bar in D, 50 μm. (PDF) [file pone.0284082.s003.pdf]

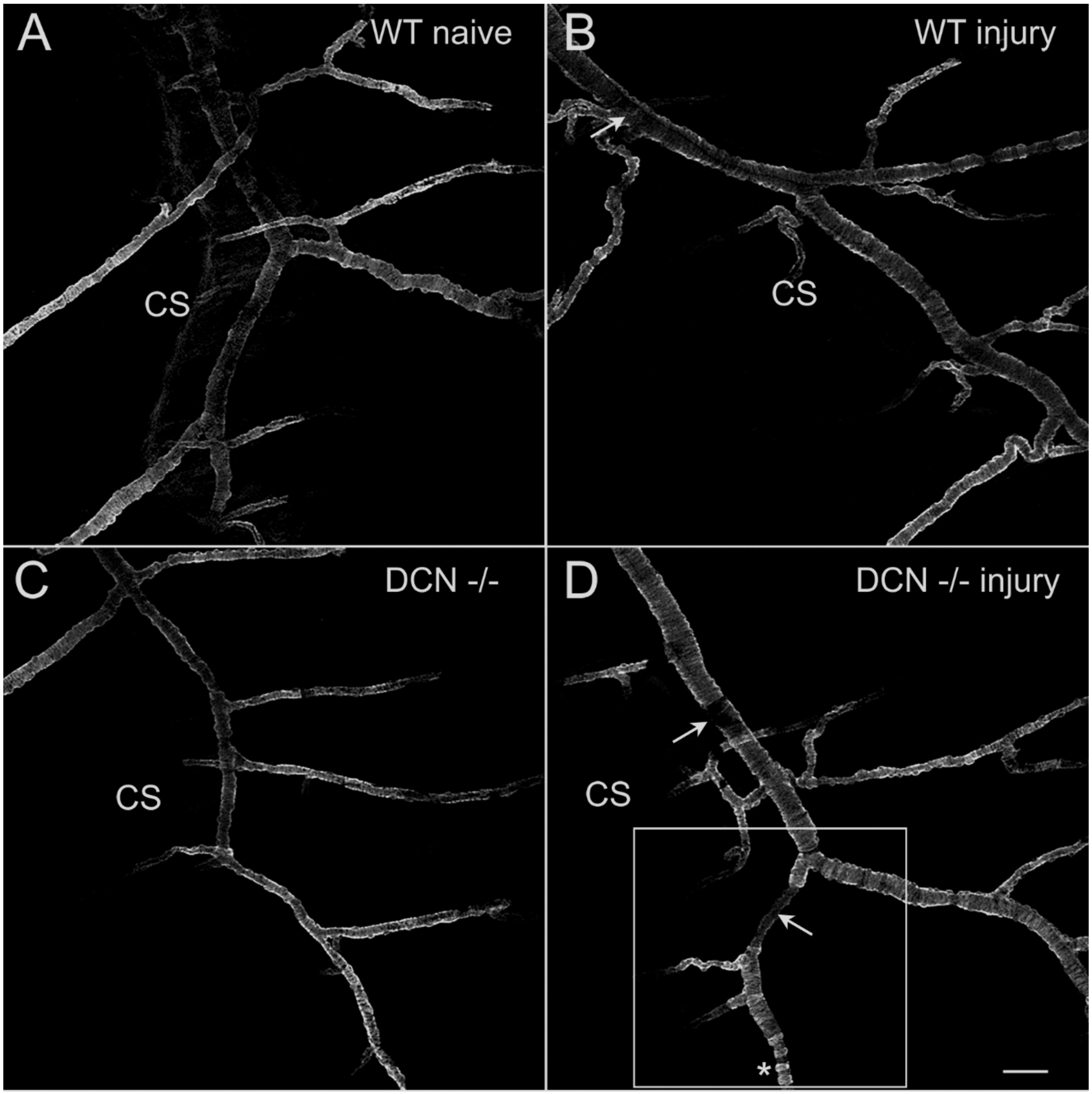

Supplemental Figure S3. Dura mater stromal and vascular responses to alkaline corneal injury. Representative immunofluorescence images of α-SMA expression from investigation area 3 taken with 20x objective. Note the loss of VSMC coverage of the blood vessels (white arrows) and acquisition of bead-like morphology by VSMCs (white asterisk). Area marked with the white square in D was imaged using 40x objective (see Figure 2, F in the manuscript) to show in more detail post injury changes in blood vessel VSMC coverage and VSMC morphology. Scale bar in D, 50 μm.
